# Improving the Precision of the Structure–Function Relationship by Considering Phylogenetic Context

**DOI:** 10.1371/journal.pcbi.0010009

**Published:** 2005-06-24

**Authors:** Boris E Shakhnovich

**Affiliations:** Bioinformatics Program, Boston University, Boston, Massachusetts, United States of America; University of California at San Diego, United States of America

## Abstract

Understanding the relationship between protein structure and function is one of the foremost challenges in post-genomic biology. Higher conservation of structure could, in principle, allow researchers to extend current limitations of annotation. However, despite significant research in the area, a precise and quantitative relationship between biochemical function and protein structure has been elusive. Attempts to draw an unambiguous link have often been complicated by pleiotropy, variable transcriptional control, and adaptations to genomic context, all of which adversely affect simple definitions of function. In this paper, I report that integrating genomic information can be used to clarify the link between protein structure and function. First, I present a novel measure of functional proximity between protein structures (F-score). Then, using F-score and other entirely automatic methods measuring structure and phylogenetic similarity, I present a three-dimensional landscape describing their inter-relationship. The result is a “well-shaped” landscape that demonstrates the added value of considering genomic context in inferring function from structural homology. A generalization of methodology presented in this paper can be used to improve the precision of annotation of genes in current and newly sequenced genomes.

## Synopsis

The author provides a novel perspective on a key problem of structural biology: the structure–function relationship in proteins. While relatedness in protein structure correlates with general description of function, attempts to use this relationship predictively are often complicated by its ambiguous nature. A structure encoded by a family of sequences may be implicated in a set of diverse functions across a variety of organisms. The author outlines an innovative approach that underlines the importance of considering genomic context when using structure-comparison methods for functional prediction.

First, the author defines two distance measures: in genomic space and in function space. Then, the author describes a landscape of functional distance based on both structural and phylogenetic relatedness.

It turns out that this landscape forms a “functional well” where proximity occurs when the structures are similar and occur in the same set of genomes. This result may have implications in future research into functional prediction. With the increasing pace of sequence deposition into databanks, this result suggests a simple way to improve functional prediction via structure homology by complementing existing methods with emerging techniques from comparative genomics.

## Introduction

Since the advent of biological data storage in digital format, researchers have struggled to define quantitative measures of comparison for sequence [1], structure [2], and function [3–5]. While proximity measures for sequence and structure are now well established, the problem of defining functional distance has been particularly daunting. Existing computational methods of describing function using ontologies are not a priori well suited for calculating functional distance [3]. However, using mostly anecdotal evidence, researchers have shown that sequences sharing key structural characteristics often display common function [6].

Nevertheless, quantitatively relating structural homology to function has been complicated by a dearth of functional distance measures and numerous examples of folds performing many unrelated functions. This many-to-many relationship between structure and function has been linked to fundamental biological processes and characteristics such as adaptation, specialization, pleiotropy, or differential regulation [7–9]. Despite these difficulties, understanding the relationship between structure and function is one of the foremost challenges of post-genomic biology [10]. Since protein function often depends on genomic context, defining predominant trends in the coalescent evolution of organisms and proteins may be instrumental in improving our understanding of the structure–function relationship [5].

## Results/Discussion

I consider the protein domain universe as the set of all structurally characterized domains [11]. I treat each domain as a structural scaffold encoded by a set of homologous sequences [4]. The power of this approach is its ability to leverage the relative conservation of function inside the structural scaffold [5] to statistically determine the relationship between structure and function. Then, using information about the distribution of the domain universe across the evolutionary tree [12,13], I hope to improve the current level of precision [4] of the structure–function relationship. Thus, for each pair of domains, I start by defining and calculating their structural, functional, and phylogenetic similarity (see Materials and Methods and [5,14–16]).

First, I define a simple but quantitative measure of functional comparison: F-score. F-score is defined as normalized Euclidian distance between GO [17] trees built from annotations of sequences coding for each structural scaffold (see Materials and Methods and [17]). Formally, *F_A,B_* = 1/*L*(Σ_*i*ε{*functions*}_(*p*_*A,i*_ − *p*_*B,i*_)^2^)^1/2^ *F_A,B_* is the functional distance between domain A and domain B, *P_[A|B],i_* is the percentage of sequences that fold into structure A or B that are annotated as function *i,* and *L* is a normalization constant that accounts for different depths of annotation on the GO. F-score measures similarity of paths on the GO tree between two sets of homologous sequences. For example, if two domains encode two sets of sequences that follow exactly the same path, F-score will be zero. On the other hand, if the sequences encoding the two domains have no common functional annotations, the F-score will be maximum.

**Figure 1:**
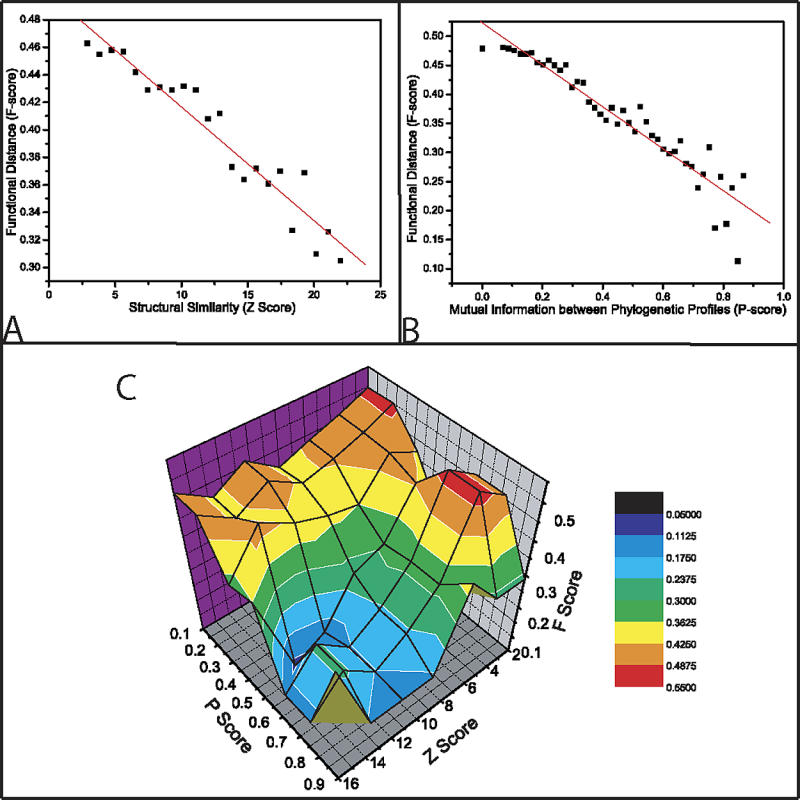
The Correlations between Z, F, and P Scores (A) The correlation between structural comparison Z-score and functional distance F-score. (Pearson's r = 0.96 and slope = 0.007.) Each bin contains at least 200 observations. It is worth noting that the average functional distance (F-score) falls from 0.48 to 0.30, only by a third during two decades of structural similarity [14]. (B) The correspondence between phylogenetic profile distances calculated using mutual information and F-score. Slope of the linear fit is 0.36, with Pearson's r = 0.96. The correlation is averaged, i.e., each data point represents a bin containing 150–200 domains, and the functional distances are averaged inside the bin [14]. (C) The landscape of functional distance with respect to Z and P scores. An average F-score is calculated for each of the 36 bins; each bin contains 100–200 observations. Since F-score is a distance metric, hotter colors represent domains that are farther away and cooler colors represent those that are closer.

Next, I set out to correlate F-score and structural similarity (Z-score calculated using DALI [2]). I expect a general correlation to hold, since previous research has shown that domains sharing key structural characteristics often perform similar functions [6,8]. Indeed, I observed a robust correlation, on average between Z-score and F-score (Figure 1A). However, the dynamic range of this correlation is small. The difference in F-score between the closest and farthest structures is only 30%. This small dynamic range most likely stems from the ambiguous relationship between structure and function.

From an evolutionary perspective, the environment is often important in defining the precise function of the sequence. Consequently, sequences appearing in the same set of genomes have been shown to perform similar functions [18]. Thus, domains with similar phylogenetic profiles should also display similar F-scores [18]. For a measure of phylogenetic similarity, I used the most commonly used mutual information (see Materials and Methods) between phylogenetic profiles of domains (P-score). Since mutual information is reflective, it is maximum when the two domains appear in the same or exactly opposing subset of genomes, and minimum when the overlap in appearance across the genomes is random. I found that P-score is a slightly better predictor of functional similarity than structural homology, with dynamic range of 50% as measured by F-score (Figure 1B). This implies that genomic context, more than constraints imposed by structure alone, may influence the precise function of the gene.

Finally, quantitative definitions of structure, function, and phylogenetic similarity allowed me to calculate the landscape of F-scores for all pairs of domains with respect to their Z and P scores (Figure 1C). Contrary to naïve expectation of smooth transitions across a small range of F-scores observed for pairwise comparisons in Figure 1A and B, I found that the combination of Z-score and P-score forms a well-shaped functional landscape with a sharp transition in F-score. This suggests that similar structures occurring in different genomes often perform dissimilar functions (see Materials and Methods). Alternatively, genes with similar structures are more likely to perform similar functions if the distribution of their orthologs on the evolutionary tree is also similar. This finding is intuitive, since genes often adapt to the environment through mutation in sequence that alters function but not structure.

The findings presented here suggest that both our understanding of the structure–function relationship and the precision of functional annotation can be greatly improved by considering structural homology in phylogenetic context. I am currently involved in work trying to improve on my naïve measure of functional similarity and assess the robustness of these results to arbitrary cutoff parameters. Furthermore, using these results it may be possible to outline a novel, optimal strategy with respect to functional annotation for the currently ongoing structural genomics projects.

## Materials and Methods

### 

Evolution is, at its core, a science of comparison. In order to study evolution, I needed to create a computational framework to represent our current body of knowledge. I chose to approach this problem from a graph-theoretic prospective in which nodes are the basic units of evolution and edges are different comparison measures. Aside from providing a unified framework, evolutionary graphs like these provide a way to organize the diverse glut of experimental data that has become the cornerstone of bioinformatics research. In the case of molecular evolution, given that domains can be functionally independent, can be expressed outside larger protein complexes in genomes, and are often rearranged through alternative splicing, I can define a domain as a good evolutionary basic unit subject to structure–function pressures. Consequently, I chose to work with annotations and comparisons of domains instead of whole proteins.

#### Structural comparison and building of PDUG.

I employed a Z-score measure of structural proximity as weight for the edges to create a protein domains universe graph (PDUG [19]). Formally, I created a graph where the nodes are the representative set of recurring structural domains identified previously by DALI [20,21], and the edges are the structural comparisons between those domains weighed by their respective Z-score. I used the above graph representation to understand the role that pressure on structure plays in the evolution of protein domains. Using this graph-theoretic paradigm, I could investigate not only the topology of the graph but also the correlation between the structural comparison graph and other dimensions of the same graph based on comparison metrics, such as function and phylogenetic proximity explained in detail below. The names of the domains used in this study are available at http://romi.bu.edu/phylo_context/domain_names.txt. The domain names refer to the DALI nomenclature as described in [22].

After I defined a PDUG, I had to populate it using sequences, so as to correlate the structures and the set of sequences that fold into those structures. I used a non-redundant database of sequences, NRDB [23]. This database straightforwardly uses sequence alignment on all known sequence databases to remove neighbors with more than 90% identity to a representative sequence, analogous to the method described above for structures. In order to map the set of recurrent domains onto sequence space, I used the now canonical BLAST [24] sequence alignment algorithm to find homologous PDUG nodes to all non-redundant sequence representatives obtained from NRDB. For every sequence in NRDB, I found the best matching sequences below 1e^-10^ threshold. Since structures from DALI are themselves devoid of sequence homologs, at most one structure is found for every non-redundant sequence from NRDB. Since each sequence is annotated with the function that it performs, this yields a mapping not only of non-redundant sequences but also of their respective functions to nodes on PDUG. The distribution of sequences from NRDB that are homologous to DALI structures is given in Figure S1.

#### Functional domain universe graph.

Since I was interested in the most general description of functionality of protein domains, I defined the function of each domain as the weighted set of functions performed by all the sequences that align to it. Thus, the functionality of the domain is represented by a probabilistic GO [25] tree. This tree is populated by taking all non-redundant sequences matching each PDUG node (as described above) and placing their functional annotations into the canonical GO. I rebuilt the whole GO tree by following all paths that led to root node from the functional annotations mined out of NRDB sequences. I increased the count of a node each time I visited it. Afterwards, all counts were turned into probabilities by normalizing the number of times that I visited each node on every level of the GO tree by the total number of times I visited that level. I ended up with a probabilistic representation of function for each structure at various levels of specificity.

Each node on PDUG now had the representative structure, the set of sequences that fold into that structure, and the set of functions performed by those sequences in the form of a probabilistic, hierarchical GO [25] tree. The benefit of representing functionality in terms of a probabilistic GO tree is that I could now compare functionality of domains by simply comparing their GO trees. If I wanted to understand the “difference” in function between two domains, I needed to take into account all functions that this structure was implicated in. For example, some sequences for a given structure may be involved in creatine phosphorylation, and others can be involved in arginine phosphorylation, as in the case of 1qh4 (Figure S2) [26].

Thus, in order to compare the GO trees, I calculated the Euclidian distance between the nodes on each level of the GO hierarchy by using Equation 1.


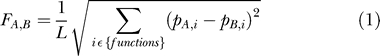


Here *F_A,B_* is the functional distance between domain A and domain B, *p_A,i_* is the percentage of sequences that fold into structure A that are annotated with function *i,* and the sum is taken over all annotated functions. F-score measures similarity of paths on the GO tree between two sets of homologous sequences. For example, if two domains encode two sets of sequences that follow exactly the same path, the F-score will be zero. On the other hand, if the sequences encoding the two domains have no common functional annotations, the F-score will be maximum. Using the above functional distance measure, I created another dimension of PDUG. In this dimension, the edges are functional comparisons between the domains and are weighed by the F-score.

#### Phylogenetic distance P-score.

Phylogenetic context (the subset of genomes where the domain is found) can have a profound effect on the function and overall evolution of that domain. Knowing this, I created another dimension of PDUG where each node was annotated with the genomes where it was present. This is done by simply BLASTing [24] the set of non-redundant sequences found in each node in PDUG against all fully sequenced and mapped genomes. This yields a mapping of structural space into genomic space. Thus, each node is annotated with a vector where columns represent the different genomes and the values are zero or one, depending on whether the domain exists in that genome.

The calculation of distance in genome space is non-trivial and is subject to all kinds of qualifications, such as relative distance between genomes on the tree [27,28]. However, I simplified the calculation by employing mutual information as a first-order approximation to distance between every two phylogenetic vectors. The distance between any two nodes in phylogenetic space is then just the mutual information between their vectors, as defined by


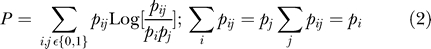


where *p_ij_* is the frequency of occurrence of all four possible combinations of presence or absence in the same genome for nodes *i* and *j,* and *p_i_* and* p_j_* are the marginal probabilities of seeing those domains in all genomes. Mutual information is a reflexive measure, insensitive to correlation or anti-correlation. Thus, mutual information will be maximal if the two phylogenetic vectors are either perfectly correlated or perfectly anti-correlated while the norm of that vector is half the length. This is a useful property for evaluating P-score, since genes that appear in a completely disparate set of genomes have been shown to perform similar functions in a process dubbed “non-orthologous gene displacement” [29]. Using this distance measure, I created the third and final dimension of PDUG where the nodes are the domains with redundant sequences and functional trees, and the edges are weighed by the mutual information measure between the phylogenetic profiles of the nodes.

#### Looking through the dimensions.

Finally, I correlated all three dimensions of PDUG, by observing the F-score between two nodes with respect to both the structural proximity and the phylogenetic distance (Table S1). The striking observation was that resolution of functional distance increases by almost 100% when considering structural proximity and phylogenetic distance over using any one of these measures alone. The combination of phylogenetic distance and structural similarity differentiates structures with close functional similarity from similar structures without functional similarity, and analogous behavior is observed for sets of domains sharing phylogenetic profiles (data not shown). The protein domains that run contrary to this trend are good candidates for investigating convergent evolution.

#### Robustness analysis.

To evaluate the robustness of the results reported in Figure 1C, I performed a jackknife analysis to evaluate the standard deviation of each data point on the graph. I sampled 60% of the data 150 times. I then gridded those points, as in Figure 1C, and then I conglomerated the results. The means and standard deviations of F-scores for each pair of Z and P scores can be accessed directly from http://romi.bu.edu/phylo_context/z_p_f_landscape_stat_sig.dat. The difference in the functional similarity score is several standard deviations away from random and is highly significant. Moreover, the overall nature of the results does not depend on the binning, or the way that the jackknife procedure is performed (data not shown).

## Supporting Information

Figure S1 The Distribution of Sequences from NRDB That Are Homologous to a Structure The data are available online from http://romi.bu.edu/phylo_context/count_seqs.out. The structures may be downloaded from the PDB directly and from the ASTRAL compendium using the domain names provided in http://romi.bu.edu/phylo_context/domain_names.txt.
(4.2 MB TIF).

Figure S2 Example of Uneven Scaffold Annotation on the Functional GO Tree (1.3 MB TIF).

Table S1 Use of Phylogenetic Distance for a Particular Structural Similarity Score Differentiates Functionally Related Proteins from Those That Are Not (449 KB TIF).

## Abbreviations

GO: Gene Ontology
PDUG: protein domains universe graph
