# Correction: Improving the Precision of the Structure–Function Relationship by Considering Phylogenetic Context

**DOI:** 10.1371/journal.pcbi.0010038

**Published:** 2005-08-26

**Authors:** Boris E Shakhnovich

DOI:  10.1371/journal.pcbi.0010009

In *PLoS Computational Biology,* vol 1, issue 1.

The Editor for this research article was incorrectly given as Philip Bourne. The Editor was Burkhard Rost, Columbia University, United States of America.

