# Supplementary figures and images for: Improving the Precision of the Structure–Function Relationship by Considering Phylogenetic Context
Source: PLoS Comput Biol. 2005 Jun 24;1(1):e9. doi: 10.1371/journal.pcbi.0010009 (PMC1183515; doi:10.1371/journal.pcbi.0010009)

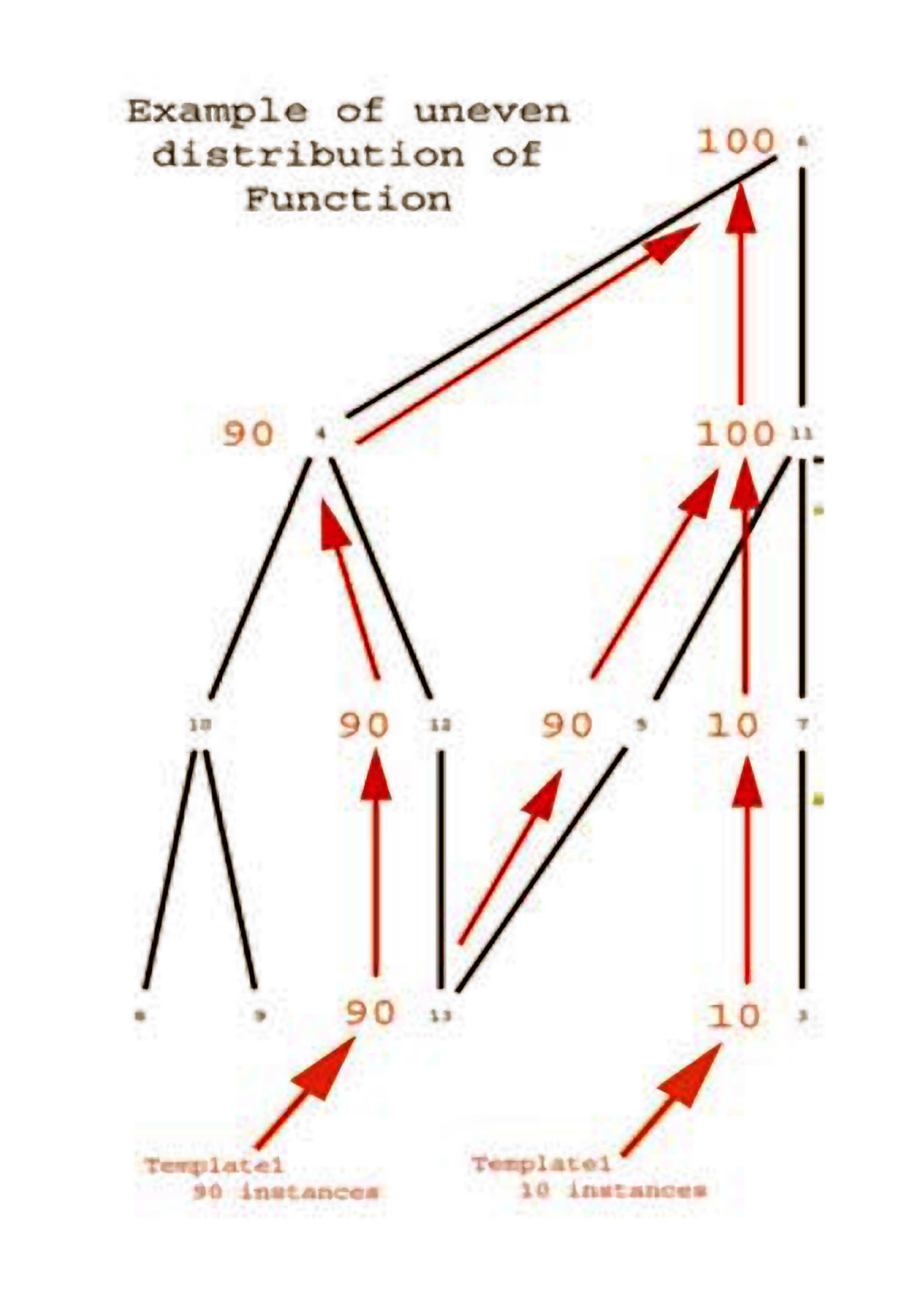

Supplement: Figure S1 — The data are available online from http://romi.bu.edu/phylo_context/count_seqs.out. The structures may be downloaded from the PDB directly and from the ASTRAL compendium using the domain names provided in http://romi.bu.edu/phylo_context/domain_names.txt. (4.2 MB TIF). [file pcbi.0010009.sg001.tif]

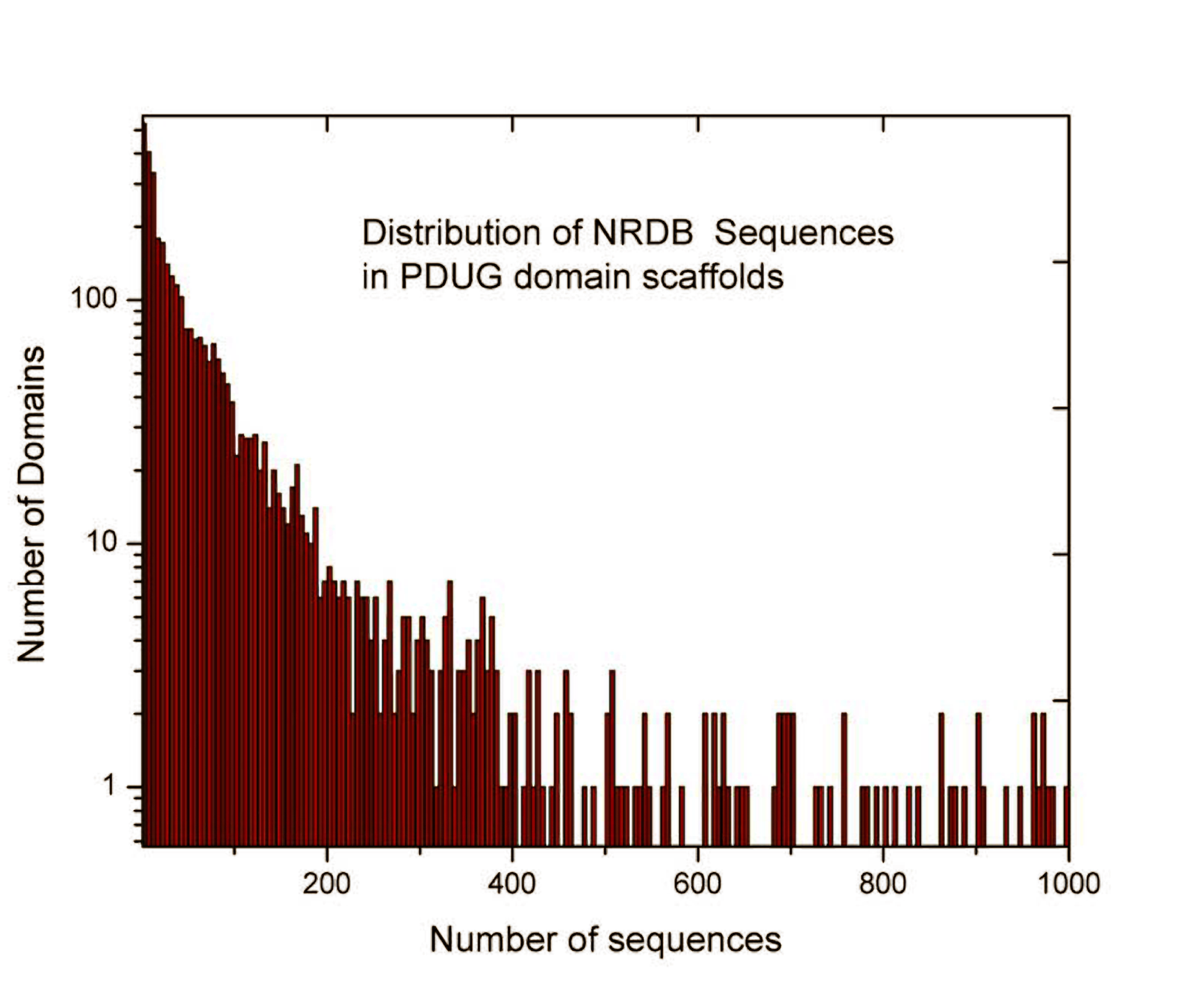

Supplement: Figure S2 — (1.3 MB TIF). [file pcbi.0010009.sg002.tif]

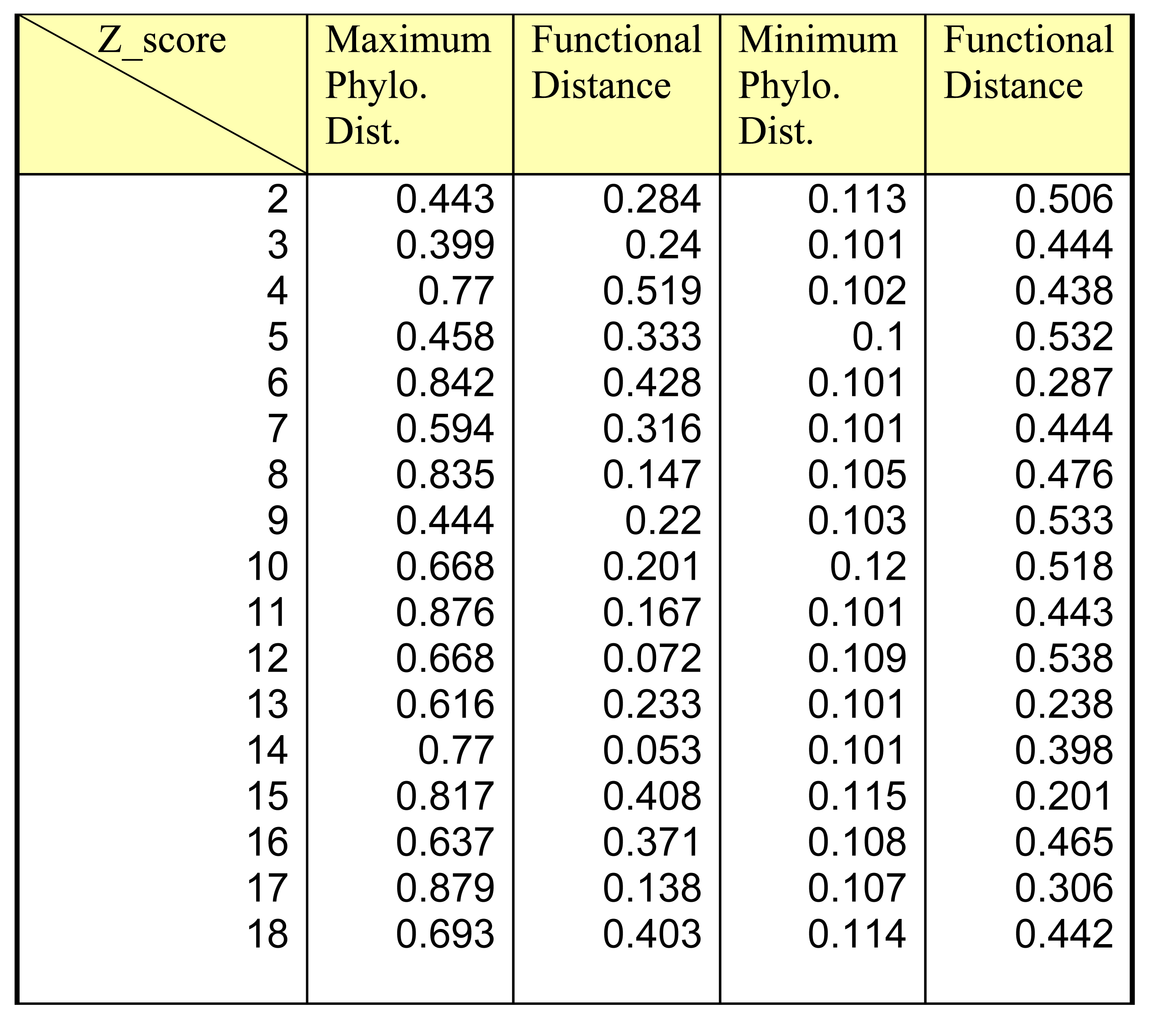

Supplement: Table S1 — (449 KB TIF). [file pcbi.0010009.st001.tif]
